# ResST-SEUNet++: Deep Model for Accurate Segmentation of Left Ventricle and Myocardium in Magnetic Resonance Imaging (MRI) Images

**DOI:** 10.3390/bioengineering12060665

**Published:** 2025-06-17

**Authors:** Abduljabbar S. Ba Mahel, Mehdhar S. A. M. Al-Gaashani, Fahad Mushabbab G. Alotaibi, Reem Ibrahim Alkanhel

**Affiliations:** 1 School of Life Science and Technology, University of Electronic Science and Technology of China, Chengdu 610054, China; dr.abdulbamahel@std.uestc.edu.cn (A.S.B.M.); profahd@gmail.com (F.M.G.A.); 2 School of Resources and Environment, University of Electronic Science and Technology of China, Chengdu 610056, China; mr.mehdhar@uestc.edu.cn; 3 Department of Biomedical Technology, College of Applied Medical Sciences, King Saud University, Riyadh 11433, Saudi Arabia; 4 Department of Information Technology, College of Computer and Information Sciences, Princess Nourah bint Abdulrahman University, P.O. Box 84428, Riyadh 11671, Saudi Arabia

**Keywords:** MRI, cardiac, left ventricle, myocardium, deep learning, segmentation models

## Abstract

The highly precise and trustworthy segmentation of the left ventricle (LV) and myocardium is critical for diagnosing and treating cardiovascular disorders, which includes persistent microvascular obstruction (MVO) as well as myocardial infarction (MI) diseases. This process improves diagnostic accuracy and optimizes the planning and implementation of therapeutic interventions, ultimately improving the quality of care and patient prognosis. Limitations of earlier investigations include neglecting the complex image pre-processing required to accurately delineate areas of the LV and myocardium (Myo) in MRI and the absence of a substantial, high-quality dataset. Thus, this paper presents a comprehensive end-to-end framework, which includes contrast-limited adaptive histogram equalization (CLAHE) and bilateral filtering methods for image pre-processing and the development and implementation of a proposed deep model for left ventricular and myocardium segmentation. This study utilizes the EMIDEC database for the training and assessment of the model, allowing for a detailed comparative analysis with six state-of-the-art (SOTA) segmentation models. This approach provides a high accuracy and reliability for the segmentation that is crucial for the diagnosis and treatment of cardiovascular disorders. The achievements of the proposed model are demonstrated by high average values of segmentation rates, such as an Intersection over Union (IoU) of 93.73%, Recall of 96.54%, Dice coefficient of 96.70%, Precision of 96.86%, and F1-score of 96.70%. To verify the generalization capability, we assessed our suggested model on five supplementary databases, which substantiates its exceptional efficiency and adaptability in a diverse environment. The presented findings demonstrate that the proposed deep model surpasses current methods, offering more a precise and resilient segmentation of cardiac structures.

## 1. Introduction

Cardiovascular illnesses remain the primary cause of mortality globally. Statistics indicate that cardiac illnesses in the United States in 2022 caused 702,880 fatalities, which is around one out of every five cases [1]. The segmentation of the left ventricle (LV) and myocardium (Myo) is crucial for cardiologists as it enables the estimation of the myocardial mass, ventricular volumes during systole and diastole, and ejection fraction. The aforementioned characteristics serve as the foundation for evaluating cardiac function and predicting outcomes in individuals with heart illnesses, including cardiac failure. This clinical condition is complicated because it involves the progression of numerous cardiac abnormalities to severe degrees [2,3].

Deep learning (DL), especially convolutional neural networks (CNNs), has achieved amazing work with medical image segmentation problems. In the past few years, research has shown that CNNs are better than traditional image processing methods because they can learn from plenty of data and pull out complex features [3,4,5]. However, even though there have been successes, there are some problems and challenges that come with utilizing deep learning in medical segmentation. Some of these are the need for huge amounts of labeled data [6], the need for a lot of computing power, and the difficulty of figuring out what the network results signify. In the active research, authors seek to enhance the precision of cardiac MRI segmentation leveraging DL models, which have demonstrated a major enhancement in the segmentation findings [7,8,9,10]. This progress should also be applicable for the segmentation of the LV and Myo, therefore opening new opportunities for cardiac diagnostics and the identification of MI and other heart disorders. The significance of creating and implementing efficient segmentation methods cannot be overstated. They provide a more precise and faster diagnosis, facilitating the start of timely treatment and enhancing the quality of life for patients. In addition, automating the segmentation process minimizes the workload on doctors and the likelihood of human mistakes [3,5,8,11,12].

In today’s medical practice, several issues are associated with diagnosing and treating cardiovascular disorders. In particular, we have to pay special attention to the identification of pathologies of the LV and Myo since these structures are vital for the proper functioning of the heart. The utilization of deep learning-based segmentation in image processing offers a robust means to automate this procedure and enhance diagnostic effectiveness [4,7,8]. Globally, cardiovascular disorders continue to be one of the leading reasons of mortality [13]. Within this substantial health concern, the requirement for the precise identification and evaluation of cardiac components, such as the LV and Myo, becomes a crucial component of preventive and therapeutic approaches. Herein comes the value of image segmentation, mostly employing DL approaches, i.e., a SOTA automated utility for medical image analysis. The methods described provide a very high rate of precision in detecting the intended structures in images and guarantee the reliability of the findings, which is crucial for both clinical practice and medical research. In the domain of medical imaging analysis, DL has emerged as a fundamental methodology [14]. The application of neural networks, namely (CNNs), in the segmentation of cardiac structures has shown substantial progress over the past decade. The combination of their computing power and data volume, as well as enhancements in learning algorithms, has resulted in models capable of precisely and automatically identifying the LV in MRI images with promising outcomes [15]. The primary benefit of DL in a segmentation task is its capability in extracting high-degree characteristics from images, enabling models to more effectively adjust to distinctive conditions and data kinds. This aspect is crucial in medical imaging, where the wide range of pathologies and anatomical features requires a considerable adaptability and generalization of the algorithms. The continual development and research of segmentation models utilizing deep learning is actively ongoing. Current research is primarily concerned with enhancing models’ precision and stability and their applicability to various kinds of medical imaging. An additional significant area of research is also focusing on the reduction in the requirements for the volume of labeled data for training models, therefore enhancing their accessibility and effectiveness in clinical practice [16].

The timely identification of pathologies and the precise quantification of parameters of cardiac structures has the potential to greatly enhance the standard of medical treatment and reduce patient risks. Therefore, developing and optimizing DL models for left ventricular and myocardium segmentation in MRI images is a pertinent and encouraging area of research, since in practical application, the precise segmentation of the LV and Myo in MRI images can enhance the diagnosis of cardiovascular disorders, optimize surgical planning, and monitor the effectiveness of the treatment. This direction necessitates a collaborative endeavor in medicine, machine learning, and computer vision to achieve optimal outcomes that serve in the treatment of patients with cardiovascular illnesses. Therefore, employing deep learning techniques to partition cardiac components in MRI images is a very promising and pertinent method that has substantial potential to enhance clinical practice in cardiology. Therefore, the objective of this research is to enhance the precision and reliability of the segmentation models, thereby enhancing the diagnostic and therapeutic precision of cardiovascular illnesses. The primary scholarly contributions of this work are as follows:

- A comprehensive end-to-end approach for the segmentation of left ventricular and myocardium structures is developed and implemented. The present work introduces a novel approach that incorporates a dual-stage image pre-processing pipeline, combining CLAHE and bilateral filtering. By taking this pre-processing stage, the quality of the input data is greatly enhanced, thereby leading to increased precision and reliability in future segmentations.
- Our work introduces ResST-SEUNet++, a DL model for segmenting cardiac and medical images, which incorporates ResNetSt (split-attention networks) as an encoder and an SE attention module in the Unet++’s decoder. To our knowledge, no prior work has employed this combination for cardiac segmentation. ResST-SEUNet++ was subjected to a thorough performance assessment and comparison to other advanced models like PAN, ResUnet, ResUnet++, Manet, Linknet, and PSPnet. Our findings demonstrated that the suggested model had a greatly superior performance when compared to existing methods in crucial rates like the IoU, Recall, Dice, accuracy, and F1-score.
- The verification of the proposed model’s exceptional precision and reliability utilizing experimental data demonstrated that the reported model exhibits an outstanding performance in cardiac LV and Myo structural segmentation tasks, as proven by the high metric values obtained from the experimental findings. These findings validate the theoretical relevance and practical applicability of the proposed methodologies in real medical situations.
- This study verified the generalization capabilities of the proposed model on five additional databases, namely the ACDC 2017 [11], Wound Closure Database [17], Kvasir-SEG [18], CVC-ClinicDB [19], and ISIC 2017 [20]. This affirmed the model’s adaptability and efficacy in a variety of datasets, illustrating its capacity to maintain a high segmentation accuracy under varying conditions.
- We incorporated an explainability scheme into the ResST-SEUNet++ model. This feature creates visual maps to show which parts of the image the model focuses on for each class. This makes the model easier to understand and interpret, helps spot mistakes, and makes its decisions more transparent and reliable.

Thereby, the core novelty of this work concisely lies in the strategic integration of a ResNeSt encoder with a Unet++ decoder augmented by squeeze-and-excitation blocks, alongside a dual-stage pre-processing pipeline. To the best of our knowledge, this integration has not been applied in previous cardiac segmentation frameworks. It leads to substantial gains in segmentation accuracy and robustness across diverse datasets. Furthermore, the incorporation of an explainability module enhances the model transparency and interpretability, reinforcing its practical value in clinical applications.

Further sections of the manuscript follow this organization: Section 2 provides an overview of existing studies on left ventricular and myocardial segmentation and discusses the relevance of deep learning methods. Section 3 describes the materials and methods, including a comprehensive explanation of the model, the datasets utilized, and the pre-processing and optimization methods. Section 4 presents the experimental results, Section 5 discusses the findings and contrasts them with prior research, and Section 6 summarizes the achieved results.

## 2. Related Works

### 2.1. Left Ventricular and Myocardium Segmentation

In this section, we review existing studies on the segmentation of the LV and Myo in MRI images, a critical task in medical diagnostics requiring high precision and reliability. Recent research has explored various methods to address this challenge, and we summarize a selection of these methods, highlighting their contributions, approaches, and shortcomings in this domain. The authors of this study [21] proposed a deep CNN for myocardial segmentation using MRI images. However, despite its high accuracy, it exhibits the following disadvantages: limited training data and a significant class imbalance, which may affect the accuracy of the segmentation of pathological regions. Most segmentation errors are observed in basal and apical short-axis slices. We should test more efficient network architectures, such as U-Net, Unet++, and ResUNet, and carry out transfer learning using large datasets to improve this method. Another key limitation of this research is the lack of consideration for patients’ unique anatomical characteristics. To enhance the segmentation precision, it would be advantageous to incorporate individual anatomical features into the analysis. The authors of [22] suggested a model that segmented the left ventricle by utilizing deep convolutional autoencoders. Nevertheless, it encounters an issue with the processing of low-resolution images. Morphological operations, among other post-processing methods, may help resolve this issue. It is also essential to mention that this model does not account for the evolution of the LV’s morphology over time. We should employ 3D networks that account for the dynamics of changes to enhance the accuracy of the segmentation. In [23], the authors suggested a model for myocardial segmentation that employs generative adversarial networks (GANs). This method permits the consideration of the variation in the myocardial morphology among patients. It is also crucial to mention studies that employ multitask learning to enhance the segmentation of the LV and Myo simultaneously [24]. A 2022 study [25] introduced the MMNet model, a multi-layer, multi-skip network that facilitates automatic left ventricular segmentation. This model exhibits a high level of segmentation accuracy without the need for the prior localization of the LV. However, the study does not address the segmentation of the Myo, despite its straightforward and effective nature, and there is room for a further enhancement of the results. To enhance the quality of the image and extract more detailed semantic information from various perceptual fields, it is necessary to implement pre-processing modules and techniques, such as CLAHE. This should enhance the outcome. In a 2023 paper [26], researchers compared the performance of an automatic LV segmentation network trained mostly on CT scan images with two whole-heart reconstruction methods. They concluded that segmenting LV volumes from respiratory motion-resolved images more closely approximates manual expert tracings of the LV endocardial boundary. This study highlights the importance of using quality images to improve the segmentation precision and can act as a foundation for the further development of DL methods in cardiac MRI segmentation. Another study examined various image pre-processing methods, model configurations utilizing a U-Net architecture, post-processing techniques, and volume estimation approaches [27]. This study provides an end-to-end analytics pipeline for an automated LV segmentation and volume estimation. However, it does not segment the Myo, which is necessary for the diagnosis of other cardiac diseases, such as MI, etc.

### 2.2. Deep Learning Models for Segmentation

Deep learning methods have become integral to modern computer vision, making it possible to solve complex image analysis problems with a high accuracy and efficiency. In particular, image segmentation, e.g., identifying and classifying objects in an image at the pixel level, has become one of the key areas of the research and application of deep models. In our current study, we plan to use various well-known pre-trained models to solve cardiac segmentation problems. This section summarizes deep model-based image segmentation methods and their applications in various fields, including medical diagnostics. We consider several deep learning architectures relevant to medical image segmentation, including U-Net [28], LinkNet [29], PSPNet [30], PAN [31], Manet [32], ResUNet [33], ResUNet++ [34], and Unet++ [35]. Among these, six models, specifically LinkNet, PSPNet, PAN, Manet, ResUNet, and ResUNet++, are employed for the quantitative comparison with the proposed method. U-Net and Unet++ are briefly reviewed to provide essential background, as they form the foundation of many modern segmentation networks, including those evaluated in this study. This overview helps contextualize the architectural advancements introduced in our proposed ResST-SEUNet++ model.

#### 2.2.1. U-Net

Computer vision has seen significant developments in recent years, especially in image segmentation. One of the fundamental achievements was the U-Net architecture, explicitly created for biomedical segmentation [28], developed by Olaf Ronneberger, Philipp Fischer, and Thomas Brox of the University of Freiburg in Germany; it features a unique “U”-shaped structure with skip connections that allows it to achieve a high accuracy when trained on a limited quantity of images. In the medical domains, where images might be distorted and are not sufficient, U-Net successfully employs data augmentation to enhance the learning from available samples. The U-Net framework has proven to be an optimal selection for applications that necessitate fast and precise segmentation on account of its speed and precision. It is a widely regarded model in medical imaging because of its effective utilization in a variety of biomedical segmentation applications, including neural structures in electron microscopy imaging and cells in light microscopy.

#### 2.2.2. LinkNet

The LinkNet model is a specialized deep neural network (DNN) that was specifically created for semantic image segmentation tasks [29]. It is famous for its high degree of efficiency and rapidity, particularly in the domains of medical image processing and autonomous driving situations. The LinkNet model is built with an encoder–decoder architecture, where the encoder is designed to extract descriptive information from the input image, while the decoder recovers semantic information, enabling precise item selection in the image. The strength of the LinkNet model is its capacity to acquire knowledge from a limited quantity of data, which renders it a compelling option for applications with limited data resources. Additionally, LinkNet owns transmission modules that provide a fast exchange of information at numerous degrees of abstraction, consequently enhancing the comprehension of the contextual aspects of an image.

#### 2.2.3. PSPNet

PSPNet, which is stands for the Pyramid Scene Parsing Network, is a robust DL network specifically structured for visual scene segmentation tasks [30]. This model is widely recognized for its capacity to effectively evaluate images at various sizes, consequently offering a more comprehensive analysis of the scene’s context. The potential strength of PSPNet lies in its utilization of a pyramid module that empowers the model to consolidate data from several degrees of spatial resolution. This methodology enables us to take into account both minute features and the general framework of the image; consequently, it greatly enhances the quality of the segmentation. Moreover, PSPNet is furnished with a context module engine that enables the model to take into account the whole parts of the image while making precise decisions about pixel classification. Thus, PSPNet is an exceedingly effective and precise image segmentation model that is extensively utilized in many domains such as autonomous driving, satellite image analysis, and medical diagnostics.

#### 2.2.4. PAN

A Pyramid Attention Network (PAN) is an enhanced architecture for semantic segmentation that incorporates Feature Pyramid Attention (FPA) and Global Attention Upsample (GAU) subsystems. The FPA algorithm incorporates contextual information from several pyramidal layers to deliver precise attention at the pixel level. To enhance low-level category localization and produce the final prediction maps, GAU employs global mean pooling. By leveraging ResNet-101, PAN reaches an exceptional segmentation precision and effective utilization of the multi-level information and contextual attention [31].

#### 2.2.5. Manet

The Multi-Scale Attention Network (Manet) is a sophisticated model created for semantic image segmentation with the primary goal of enhancing the accuracy of the object recognition. The framework employs two primary attention mechanisms, namely Position-Wise Attention and Multi-Scale Fusion Attention. These methodologies facilitate the effective analysis of spatial relationships between pixels and the incorporation of both local and global context dependencies. The Manet model has demonstrated a robust performance in medical segmentation tasks, including liver and tumor segmentation, with high average Dice coefficients [32].

#### 2.2.6. ResUNet

ResUNet is a DL model developed for segmenting semantic images. It integrates the advantages of two architectures, namely the ResNet CNN and the U-Net network. ResNet is famous for its capacity to acquire knowledge from complex mathematical structures while circumventing the issue of gradient degradation. ResUNet integrates the fundamental components of ResNet, which provide the effective extraction of image characteristics at various degrees of abstraction, along with the U-Net architecture for precise object segmentation in digital images. In numerous applications that need a high precision of image segmentation, including medical image processing and object recognition in satellite images, the ResUNet architecture has proven to be efficient [33].

#### 2.2.7. ResUNet++

In the area of medical image segmentation, ResUNet++ is a sophisticated structural framework that is derived from an enhanced version of U-Net. This system integrates ResNet components and an attention mechanism to enhance the computational efficiency of handling intricate and diverse medical images. The essential characteristics of this architecture include resident blocks to enhance the gradient flow, attention mechanisms to concentrate on prominent areas of the image, multi-scale features to encapsulate the context at different resolution degrees, and enhanced skip connections to optimize the utilization of information from preceding layers. Compared to previous models, ResUNet++ has demonstrated an excellent performance in medical image segmentation tasks, which makes it a valuable instrument for clinical applications and automated diagnostics [34].

#### 2.2.8. Unet++

Unet++ is an enhanced version of the U-Net architecture that was structured for semantic image segmentation. Unet++ achieves an enhanced segmentation precision by utilizing dense pass paths and cross-convolutions, which facilitate information flow across network levels. The framework has many decoders matched with encoders to enhance the restoration of intricate features in images. The Unet++ model has proven to have an exceptional performance in medical imaging and other domains that demand accurate segmentation, including satellite image processing and biomedical research [35]. Thus, the application of such DL in LV and Myo segmentation is a very promising and growing research domain. The latest advancements in techniques and models have shown to be highly accurate and efficient. However, they also introduce various obstacles, including the requirement to boost the image quality, to make use of advanced DL models, and to include medical knowledge. Subsequent investigations should prioritize enhancing these aspects to provide more precise and efficient segmentation for clinical practice.

## 3. Materials and Methodology

**Figure 1:**
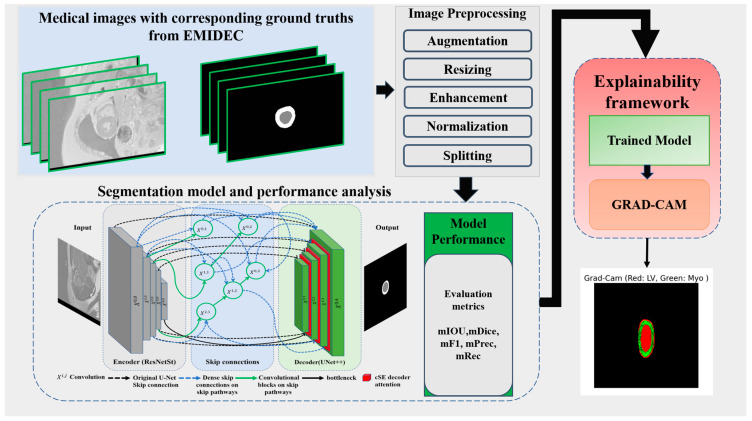
Proposed deep learning-based left ventricular and myocardium segmentation framework.

Our methodological framework, depicted in Figure 1, presents a comprehensive pipeline for medical image segmentation and interpretability. The process begins with EMIDEC images and their corresponding ground truth annotations. These images undergo a series of pre-processing steps, including data augmentation, resizing, contrast enhancement, normalization, and dataset partitioning. The pre-processed data are then fed into a segmentation network that integrates a ResNeSt-based encoder with a Unet++ decoder enhanced by channel squeeze and excitation (cSE) attention mechanism to train and perform semantic segmentation of three classes: background (BG), LV, and Myo. Model performance is quantitatively assessed using standard evaluation metrics such as mIoU, mDice, mF1–score, mPrecision, and mRecall. To enhance model interpretability, a Grad-CAM-based explainability framework is employed, enabling the visualization of class-specific feature activations in the segmented outputs.

### 3.1. Datasets Description

This section presents an elaborate analysis of the main databases utilized for training, performance evaluation, and testing of the generalization ability of the proposed model.

#### 3.1.1. EMIDEC 2020

**Figure 2:**
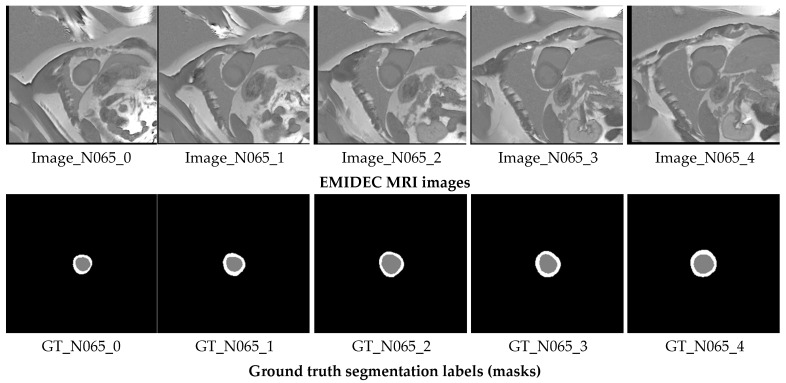
Short-axis MRI slices of a N065 normal patient alongside their corresponding GT segmentation labels. The gray label represents the LV, while the white label corresponds to the Myo.

EMIDEC 2020 is a new publicly available DE-MRI database (https://emidec.com/, accessed on 3 June 2024) including 150 clinical cases, along with relevant clinical information [36]. The training set consists of 100 cases with ground truth (GT) masks, including 33 normal and 67 pathological cases. The test set comprises 50 cases, 17 normal and 33 pathological, without GT masks. Since the official test set does not provide ground-truth annotations, it cannot be used for computing evaluation metrics. To address this, we re-partitioned the labeled training data into new training, validation, and test subsets to ensure a fair and measurable assessment of model performance. The original test set, lacking ground truth, was instead utilized for supplementary visual analysis as part of an additional qualitative validation study to further demonstrate the model’s practical applicability. This dataset has been demonstrated to be capable of being utilized to develop methodologies to automatically classify various relevant regions (LV—left ventricular, Myo—myocardium, MI—myocardial infarction, and MVO-persistent microvascular obstruction) from a series of short-axis DE-MRIs covering LV and then quantify MI estimates in absolute value (mm^3^) or as a percentage of the myocardium [37]. The quantity of images varies for each patient, from 5 to 10 short slices encompassing the left ventricle (LV) area. Expertly drawn segmentation labels for these regions were provided for each MRI slice. Figure 2 illustrates an instance of the original short-axis MRI series alongside their respective GT annotations for an N065 normal patient.

#### 3.1.2. ACDC 2017

ACDC 2017 database is a specialized medical dataset used to develop and evaluate cardiac MRI segmentation algorithms [11] (Automated Cardiac Diagnosis Challenge (ACDC), https://www.creatis.insa-lyon.fr/Challenge/acdc/scientificInterests.html, accessed on 6 June 2024). This specialized medical dataset, collected at the University Hospital of Dijon, includes information on 150 patients divided into five groups reflecting different pathological conditions of the heart muscle. It provides data on the normal heart as well as patients with MI, dilated cardiomyopathy (DCM), hypertrophic cardiomyopathy (HCM), and right ventricular anomaly. Images obtained using a 1.5 Tesla scanner cover the phases of end-diastole and end-systole, presented as short-axis cine MRI sequences. Experienced cardiologists manually annotated each image, resulting in segmentation masks for the LV, right ventricle (RV), and Myo. The ACDC 2017 database contains 1902 training and 1076 testing images and is widely used in developing segmentation algorithms, clinical research, and comparative analysis of cardiac images. Its use helps address anatomical and pathological variability and provides high-quality annotations for training robust cardiac MRI segmentation models.

#### 3.1.3. Wound Closure Database

To evaluate the proposed model’s capacity for generalization, we utilize the wound closure progress database available at the following link: (https://datadryad.org/stash/dataset/doi:10.25338/B84W8Q, accessed on 10 June 2024) [17]. This dataset contains 256 images showing the progress of wound healing in mice over 15 days after wounding. This dataset provides a valuable resource for medical biology and medical photography researchers, allowing the study and analysis of wound healing processes at the molecular and tissue level. The images in this database do not contain labels, so we used the Labelme python package [38] in the Python programming language to annotate them. After annotating the data, we created the corresponding masks correctly, and then we augmented the data using techniques from the albumentations python package [39], which allowed us to generate 2000 images. Implementing this methodology enabled us to broaden and diversify our dataset, consequently augmenting its size and variety. Thus, this action enhanced the precision of evaluating the generalization capability of our model. Analyzing the findings from this database allowed us to enhance our study and verify the robustness of the suggested approach under different circumstances and source data.

#### 3.1.4. Kvasir-SEG

Our evaluation of the suggested model’s applicability was conducted utilizing the Kvasir-SEG database [18]. The Kvasir-SEG database, which is available at https://datasets.simula.no/kvasir-seg/ (accessed on 12 June 2024), is specifically prepared for polyp segmentation. It comprises 1000 polyp images together with their matching reference masks. To enhance the training process as well as the stability of the model, we implemented data augmentation techniques, enabling us to augment the number of input images to 2000.

#### 3.1.5. CVC-ClinicDB

The Colon Video Capsule Endoscopy Database (CVC-ClinicDB) [19] is a comprehensive and proprietary dataset specifically created for research in the realm of polyp recognition and semantic segmentation tasks. It may be accessed at https://polyp.grand-challenge.org/CVCClinicDB/ (accessed on 14 June 2024). A valuable resource for developing and assessing automated medical image analysis systems, especially for detecting polyps and other colon diseases, this database comprises 612 images accompanied by appropriate annotations given by specialists. The inclusion of a ground truth for each image in CVC-ClinicDB guarantees a high degree of accuracy and dependability of the data. The database’s primary characteristics of diverse cases, annotations, and imaging conditions render it an essential instrument for the training and evaluation of machine learning (ML) and computer vision (CV) algorithms in the realm of medical diagnostics. CVC-ClinicDB is expediting substantial advancements in the development of automated systems for diagnosing and detecting colon disorders at an early stage. This, in turn, has the potential to enhance clinical results and decrease mortality rates associated with colorectal cancer.

#### 3.1.6. ISIC 2017

To thoroughly assess the proposed model’s generalization performance, we also validated it on the ISIC 2017 (International Skin Imaging Collaboration, https://challenge.isic-archive.com/data/#2017, accessed on 16 June 2024) dataset [20]. This dataset comprises 2000 images together with their matching masks, enabling an objective assessment of the precision of skin lesion segmentation. The ISIC 2017 is a widely recognized and comprehensive database in the realm of dermatological diagnostics, offering high-quality annotated images for developing and testing segmentation algorithms. Incorporating this dataset into the validation procedure of our model offers a rigorous assessment of the model’s capacity to generalize and prove its performance in real-world clinical situations.

### 3.2. Pre-Processing

#### 3.2.1. Cardiac MRI Enhancement

This work examined the application of various image pre-processing techniques to enhance the quality of the raw images. The techniques referred to are CLAHE [40] and the bilateral filter approach [41]. The CLAHE algorithm achieves exact image histogram equalization by taking into consideration contrast patterns in various regions of the image. Post-application of CLAHE to enhance contrast, images frequently exhibit heightened noise as a result of the increased contrast. Taking into account this concern, we have chosen to implement a bilateral filter, which enables the preservation of distinct borders of objects in the image, while simultaneously decreasing the amount of noise. The maintenance of sharp borders is of greatest significance in image segmentation tasks as it enables more precise identification of items in the image and enhances the quality of subsequent analysis. Hence, the integration of CLAHE with a bilateral filter achieves a harmonious equilibrium between enhanced contrast and reduced noise, greatly enhancing the quality of image pre-processing in the domain of image segmentation.

#### 3.2.2. Cardiac MRI Augmentation

Image augmentation is the process of modifying existing images to generate fresh data variants for training machine learning or deep learning models. By boosting the diversity of the training dataset, this approach significantly enhances the model’s generalization ability [42]. Image augmentation involves the application of several transformations, including rotations, reflections, scaling, adjusted brightness and contrast, introduction of noise, and other similar operations. The aforementioned changes enable the creation of several image versions while maintaining their semantic information. By employing random rotation or reflection, it is possible to generate fresh iterations of images that preserve the spatial information of objects featured on them.

**Figure 3:**
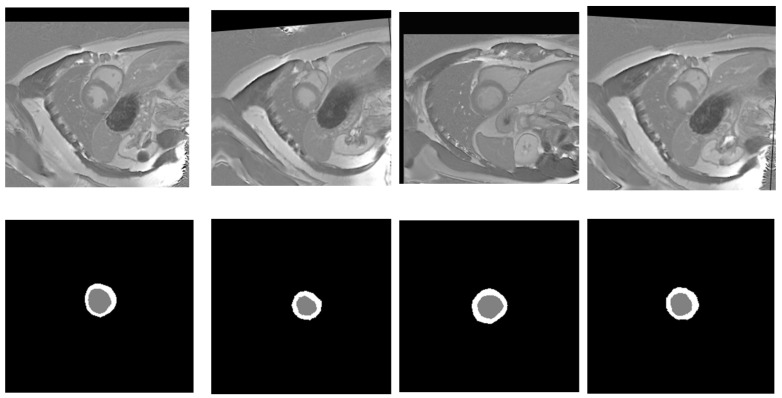
Random samples of augmented images (**top row**) alongside their ground truth labels (**bottom row**). Gray color denotes the LV, while white signifies the Myo.

Image augmentation is widely used in computer vision to improve models’ performance and generalization, especially when there is limited training data. This technique also helps models learn under different lighting conditions, orientations, and background noise, making them more robust and reliable in real-world applications. Since the source training data contains only 33 normal cases with 235 original images, we applied data augmentation techniques [43] to cardiac MRI images to augment the training data and improve the model training. It is a crucial step that allows us to increase the variety of data for training the model. To enhance the robustness and generalizability of the model while maintaining anatomical realism in cardiac MRI data, we adopted a carefully designed data augmentation strategy. A horizontal flip was applied with a probability of 0.5, introducing left–right symmetry without violating the anatomical integrity of the images. Mild rotations up to 5 degrees were allowed with a 30% chance to simulate natural variations in patient positioning during scanning. In addition, small-scale geometric transformations, including slight shifts (up to 3%) and zooms (up to 5%) combined with the same modest rotation limit, were applied with a 50% probability to introduce controlled spatial diversity. To simulate minor variations in scanner acquisition settings, subtle changes in brightness and contrast, limited to 5% in either direction, were introduced with a 30% probability. This augmentation setup prioritizes clinically plausible transformations, ensuring the anatomical consistency of cardiac structures while still promoting sufficient variation to aid in model generalization. These augmentation methods generate various images, which allows the model to learn under different conditions and enhances its generalization ability. This augmentation process results in generating 3000 images in total. Figure 3 shows different examples of augmented images with their corresponding masks.

### 3.3. Proposed Model

**Figure 4:**
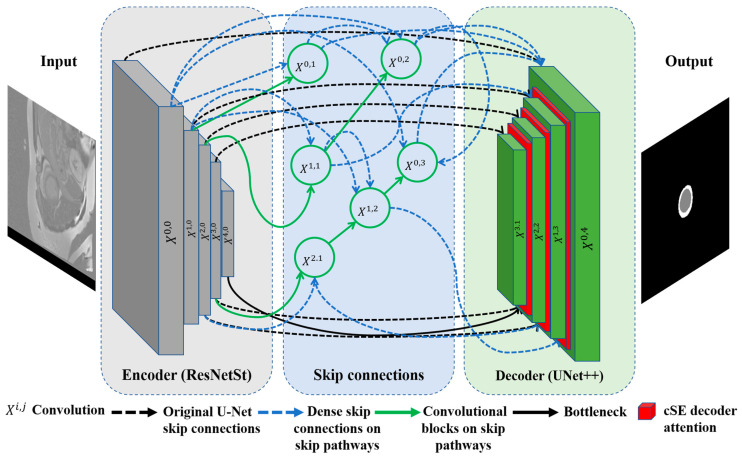
Proposed ResST-SEUNet++’s model architecture.

**Figure 5:**
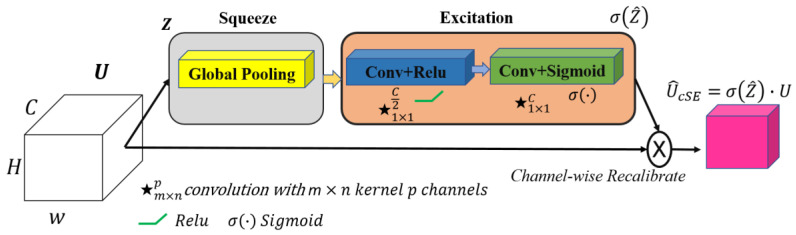
The squeeze and excitation channel attention block. The input feature map U, where C denotes the number of channels, undergoes global average pooling, followed by two 1×1 convolutional layers with ReLU and sigmoid activations to produce channel-wise scaling factors. These factors recalibrate U via an element-wise multiplication, yielding the output feature map ${\hat{U}}_{cSE}$ with enhanced channel dependencies. Note: ★ denotes a convolution operation.

In our quest to improve architectures for medical image segmentation, we present a novel model that combines the benefits of UNet++ [35] with the power of the ResNeSt (split-attention networks) encoder [44] and cSE decoder attention blocks [45]. The effectiveness of the ResNeSt encoder lies in its ability to divide attention. It uses a channel-splitting mechanism to improve feature representation and allows the model to pay attention to different aspects of the image, which is especially significant for accurate segmentation. For further enhancement of the model performance, we incorporate channel SE attention blocks into the network’s decoder. In our study, we also evaluate its performance in comparison to concurrent spatial and channel squeeze and excitation (scSE) decoder attention blocks [46]. These blocks integrate the squeeze and excitation (SE) [45] channel attention with spatial attention. This integration enables the model to flexibly adjust to various situations and enhances the precision of segmentation. Thus, we incorporate ResNeSt and SE decoder attention blocks into the design of UNet++. It enables us to preserve the advantages of UNet++ in terms of context and local information, while enhancing them with ResNeSt and SE mechanisms. The result is an efficient and accurate model for segmenting the left ventricle and myocardium in MRIs. The architecture of the proposed model is illustrated in Figure 4. The gray blocks in Figure 4 correspond to the encoding part of the model, while the green ones correspond to the decoding part. The dashed black arrows represent standard skip connections, while the dashed blue arrows denote dense skip connections, and the solid green arrows indicate convolutional blocks on skip pathways. The X^i,j^ blocks represent convolutional operations, and the red blocks correspond to the cSE attention mechanism, which is further visualized in detail in Figure 5.

### 3.4. Loss Function

The loss function is crucial in image segmentation problems since it assesses the difference between predicted and actual segmentation. This section addresses the loss function utilized to optimize the proposed model, which is a Multiclass Dice Loss as described in Equations (1)–(3):

(1) $${Dice}_{c}=\frac{2\sum_{i}p_{i,c}g_{i,c}+smooth}{\sum_{i}p_{i,c}+\sum_{i}g_{i,c}+smooth+\epsilon }$$

(2) $${Average}_{Dice}=\frac{1}{C}\sum_{c=1}^{C}{Dice}_{c}$$

(3) $$Multiclass_{Diceloss}=1-\frac{1}{C}\sum_{c=1}^{C}{Dice}_{c}\;$$

where C is the number of classes, $p_{i,c}$ is the predicted probability for the i-th pixel or (voxel) belonging to class *c*, $g_{i,c}$ is the ground truth label for the i-the pixel or (voxel) for class c, smooth is the smoothness constant to improve training stability and is set to 0.0 by default, and ϵ is a small constant (set to $1\times 10^{-7}$) added for numerical stability. The implementation of this loss offers advantages such as promoting precise object localization in images. This contributes to enhancing the overall performance of the model in image segmentation tasks.

### 3.5. Evaluation Metrics

In the overall scheme of semantic segmentation, we evaluated the model’s performance utilizing the following metrics as outlined in Equations (4)–(11): average Intersection over Union (IoU) coefficient, average Dice coefficient, F1-measure, precision, and Recall. Each of these metrics is an essential instrument in evaluating the model’s quality, enabling us to qualitatively and statistically evaluate its capacity to recognize objects in images and perform their segmentation with precision. A comprehensive examination of these measurements enables us to determine the shortcomings and strengths of the model, which is crucial for subsequent enhancement and optimization.

(4) $${IOU}_{gr}=\frac{y_{gr}^{true}\;\cap \;y_{gr}^{pred}}{y_{gr}^{true}\;\cup \;y_{gr}^{pred}}=\frac{{TP}_{gr}}{{TP}_{gr}+{FP}_{gr}+{FN}_{gr}},$$

(5) $${IOU}_{LV}=\frac{y_{LV}^{true}\;\cap \;y_{LV}^{pred}}{y_{LV}^{true}\;\cup \;y_{LV}^{pred}}=\frac{{TP}_{LV}}{{TP}_{LV}+{FP}_{LV}+{FN}_{LV}},$$

(6) $${IOU}_{Myo}=\frac{y_{Myo}^{true}\;\cap \;y_{Myo}^{pred}}{y_{Myo}^{true}\;\cup \;y_{Myo}^{pred}}=\frac{{TP}_{Myo}}{{TP}_{Myo}+{FP}_{Myo}+{FN}_{Myo}},$$

(7) $$meanIOU=\frac{{IOU}_{gr}+{IOU}_{LV}+{IOU}_{Myo}}{3},$$

(8) $$meanPrecision=\frac{1}{3}\sum_{c\in \{gr,LV,Myo\}}\frac{{TP}_{c}}{{TP}_{c}+{FP}_{c}},$$

(9) $$meanRecall=\frac{1}{3}\sum_{c\in \{gr,LV,Myo\}}\frac{{TP}_{c}}{{TP}_{c}+{FN}_{c}},$$

(10) $${Dice}_{c}=2\times \frac{{Precision}_{c}\times {Recall}_{c}}{{Precision}_{c}+{Recall}_{c}}={F1\text{-}Score}_{c},$$

(11) $$meanDice=\frac{1}{3}\sum_{c\in \{gr,LV,Myo\}}D{ice}_{c}=meanF1\text{-}Score.$$

where $y_{true}$ is the ground truth; $y_{pred}$ is the prediction; and ${IOU}_{gr},\;{IOU}_{LV},\;and\;{IOU}_{Myo}$ are the IOU metrics of the ground truth, left ventricular, and myocardium, respectively.

## 4. Experimental Results

In this section, we outline the primary findings obtained during our experiments. It includes outcomes from the data pre-processing stage, segmentation, and other key findings.

### 4.1. Training Environment and Hyperparameters

The training of our model was performed utilizing an NVIDIA A100 80GB PCIe GPU graphics card, Python 3.12.3, and the Pytorch 2.3.0 environment. These technologies offer sophisticated functionalities for the development and training of DL models, therefore enhancing the efficiency and flexibility in our experiments. The essential hyperparameters that were utilized in our training experiments include the Adam optimizer [47] with an initial learning rate (lr) of 0.0001, a batch size of eight, and 100 epochs. We selected these parameters depending on preliminary experiments to optimize the efficacy of the parameter updating and ensure an adequate number of iterations in the time of the model training.

### 4.2. Results of Medical MRI Image Enhancement and Analysis for Cardiac Segmentation

In this subsection, we detail image enhancement outcomes that were achieved utilizing CLAHE and the bilateral filtering techniques. These two methods have demonstrated significant utility in cardiac segmentation utilizing DL models. In medical imaging processing, especially in cardiac image segmentation, image enhancement techniques are essential. The process of cardiac segmentation is an essential component for the diagnosis and treatment of cardiovascular disorders. We do apply the above techniques to enhance the images, because it enables DL models to more precisely identify the boundaries and structures of the heart and therefore enhances the diagnostic precision and reliability. CLAHE is an image contrast enhancement method that applies an adaptive histogram equalization locally to small blocks of an image rather than globally. This approach improves the local detail while preventing an excessive noise amplification in homogeneous regions through a contrast-limiting mechanism. The method divides the image into tiles, applies the histogram equalization in each tile, and limits the maximum contrast in each block to avoid the over-amplification of the image. Neighboring blocks are then interpolated to ensure smooth transitions between regions. CLAHE is particularly useful in medical imaging and computer vision tasks, improving the visibility of fine details in low-contrast images while controlling noise amplification.

**Figure 6:**
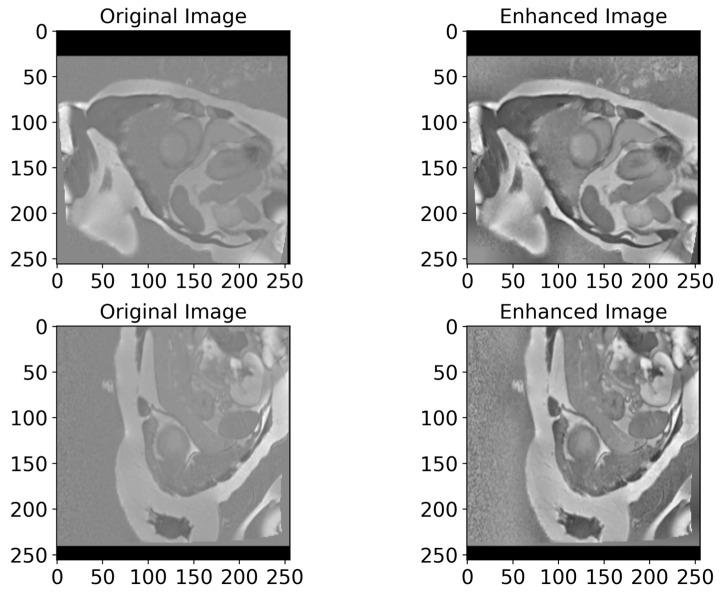
A visualized comparison of original images with enhanced images. The left column presents the original MRI images of the heart, while the right one shows the enhanced images processed utilizing CLAHE and bilateral filtering methods. Visually, the enhanced images demonstrate an increased contrast and clarity, which leads to a more accurate heart segmentation. These visible improvements give DL models the ability to more accurately highlight critical structures and borders of the heart, which in turn improves the diagnosis quality.

**Figure 7:**
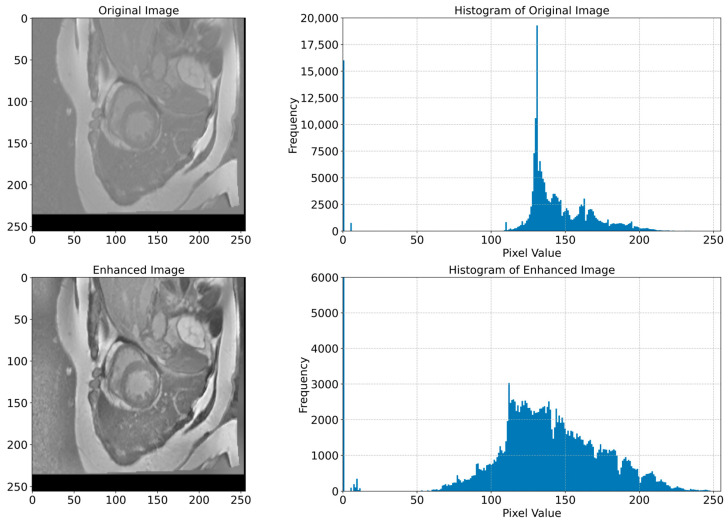
Original and enhanced images with their corresponding histograms. The top row shows the original MRI image of the cardiac region, and its histogram shows the distribution of pixel values. The bottom row shows the enhanced image and its histogram, which shows the redistribution of pixel values that resulted in an increased contrast and improved visibility of cardiac structures. This redistribution is necessary for a better recognition and segmentation of cardiac structures, which is crucial for the application of DL models in the segmentation tasks.

In Figure 6, we present examples of original images and matched enhanced images. They highlight how CLAHE and bilateral filtering techniques enhanced the image quality by making cardiac structures clearer and more contrasting. In Figure 7, the original and enhanced images, along with their histograms, are presented. The histograms illustrate how these two techniques redistribute pixel values, therefore increasing the contrast and enhancing the visibility of crucial details. This redistribution enables the better recognition and segmentation of cardiac structures, which is critical when applying DL models in medical imaging. As we can see from Figure 6 and Figure 7, the applied image enhancement techniques significantly improved the quality of the input data to DL models, which results in a more accurate heart segmentation and, therefore, better results in the diagnosis and treatment of cardiovascular illnesses. After the initial pre-processing, we performed a normalization process on all the images, which gives us the ability to convert every pixel value from the range of [0, 255] to the range of [0, 1]. This ensures an optimal performance of the DNN because normalized values constantly contribute to the stability and efficiency of the model training. Additionally, we resized all images to 256 × 256 pixels, because standardizing image sizes are crucial to ensure the consistency of the input data, which in turn improves the training of our model and simplifies data processing.

### 4.3. The Performance of the Proposed Model

In this section, we describe the segmentation findings achieved using the proposed model. We utilized the MRI image data from the Emidec dataset to evaluate our model’s performance. Once the pre-processing is complete, the data is fed into the proposed LV and Myo segmentation model. It demonstrated a high performance and achieved an average Intersection over Union (MIoU) of 93.73%. Additionally, it reached high average values of other metrics, including a Recall of 96.54%, a Dice of 96.70%, a precision of 96.86%, and an F1-score of 96.70%, which are presented in Table 1. We utilized these metrics to evaluate the model performance comprehensively, because these metrics give us the opportunity to evaluate both the overall quality of the segmentation and the ability of the model to distinguish between different structures precisely.

In Table 1, the high rates of the MIoU and other metrics verify the ability of our model to segment cardiac structures in MRI images precisely. The outcomes obtained demonstrate that the proposed model is applicable for use in clinical practice to improve diagnostic accuracy. Additionally, such findings highlight the potential of the DNN in the segmentation of medical images, where the high precision and robustness of segmentation models can significantly affect the quality of medical treatments. The successful implementation of this model could lead to significant enhancements in the early diagnosis and monitoring of cardiovascular illnesses as well as improve the treatment effectiveness and patient outcomes.

**Figure 8:**
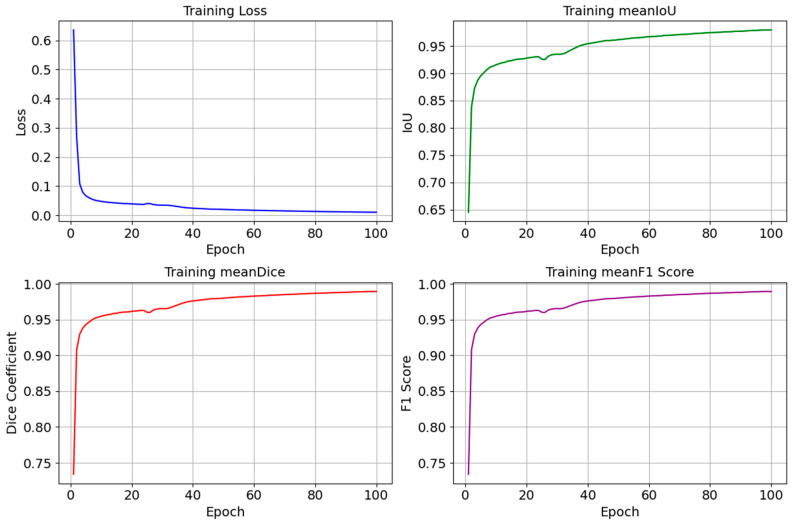
Graphs of changes in the loss (Loss), Intersection over Union (IoU), Dice coefficient (Dice), and F1-score during the model training process. Smooth curves show a stable learning process, a decrease in loss, and an increase in the IoU, Dice, and F1-score values, indicating an improvement in the model performance.

We monitor changes in metrics such as the loss, Intersection over Union (IoU), Dice coefficient, and F1-score during the model training to understand how well our model learns and how effectively it can generalize to new unseen data. In DL, loss measures the difference between the predicted labels and the actual labels, and when it decreases, it refers to an improvement in the model. We also utilized the IoU, the Dice coefficient, and the F1-score to evaluate the quality of the segmentation outcomes, where high values of these rates indicate an accurate match between the predicted and actual masks. In Figure 8, we illustrate the graphs that represent the loss, IoU, Dice, and F1-score curves. Their changes’ smoothness indicates a stable learning process. The smooth curves without sharp fluctuations in Figure 8 tell us that our model adapts well to the data and does not overfit. When analyzing these curves, the decrease in the loss curve reaches ~0.011, and the simultaneous increase in the IoU reaches ~0.98%, while the Dice coefficient and F1-score values reach ~0.99%. All these values verify the progress in the model training and its ability to perform the segmentation task more precisely.

**Figure 9:**
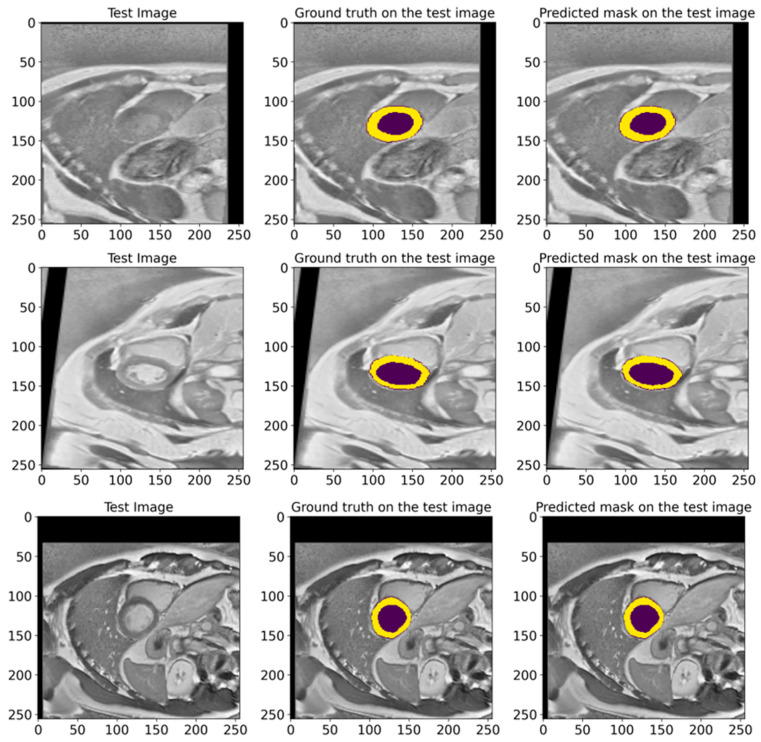
Representative examples from the EMIDEC test set, showing the GT masks and the corresponding predictions made by our model. The deep violet areas represent the LV, while the yellow areas correspond to the Myo.

Figure 9 illustrates various examples from the test dataset with the corresponding actual masks generated by the experts and the predicted masks produced by our model. Examples demonstrate the segmentation accuracy of the model. A comparison of the actual and predicted masks shows that the proposed model successfully identifies the significant structures of the cardiac region, including the left ventricle and myocardium. The model demonstrates a high accuracy even in complex cases where the boundaries of the structures are less clear. These visual results confirm the effectiveness of the developed pre-processing methods and model architecture in improving the quality of the automatic segmentation of medical images. Our proposed heart segmentation model based on deep neural networks and image enhancement demonstrated a high efficiency and accuracy. It confirms its potential for use in clinical practice and contributes to improving methods for diagnosing cardiovascular diseases.

### 4.4. Comparison with Other State-of-the-Art Models

**Figure 10:**
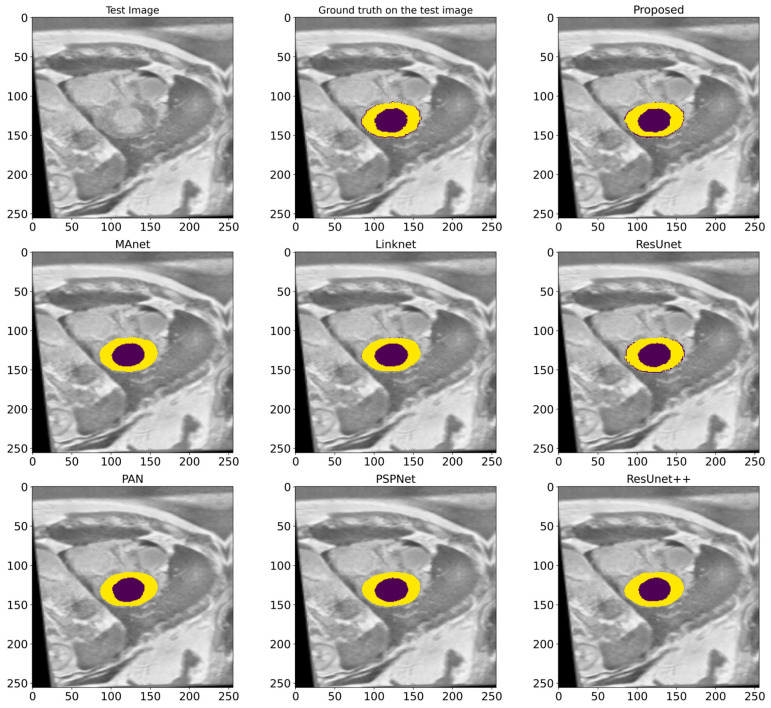
A visualized comparison of predictions of different deep segmentation models. The LV is marked in the deep violet color, and the Myo is highlighted in yellow.

**Figure 11:**
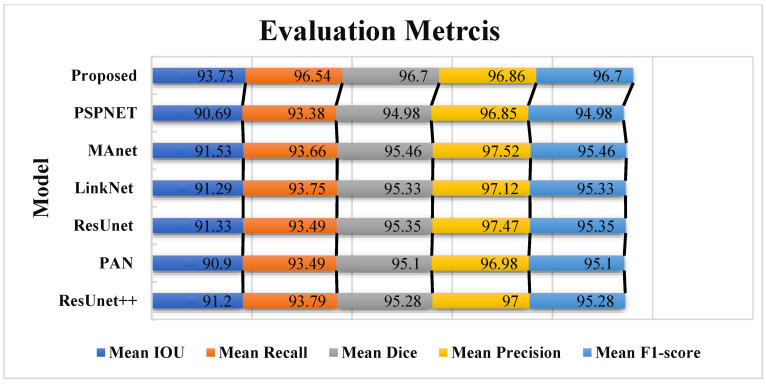
The comparative visualization of the evaluation metrics of the proposed model and six other SOTA deep segmentation models.

In this work, a comparative evaluation of the performance of the proposed model with six other SOTA deep segmentation models was carried out to demonstrate its superiority over its analogs. Figure 10 provides a qualitative comparison of segmentation results produced by various models against the ground truth. The proposed model demonstrates the highest visual accuracy, closely aligning with the manual annotations for both the LV and Myo regions. ResUnet also performs well, showing smooth and spatially coherent boundaries, although with slightly less precision than the proposed model. In contrast, models such as PAN, PSPNet, and ResUnet++ tend to over-segment the Myo area, while others like MAnet and LinkNet display a moderate agreement with the ground truth. These visual results support the effectiveness of the proposed architecture in capturing detailed anatomical structures more reliably than existing approaches. Table 2 provides more evaluation metrics, such as the Recall, Dice, precision, and F1-score, that allow for a thorough comparison of the performance of different models. The visualization of these metrics is presented in Figure 11, which enables us to demonstrate their comparison. The analysis of the results showed that the proposed model not only outperforms other modern models in most metrics but also demonstrates stable results on various datasets, which indicates its versatility and reliability. Table 2 shows that our model demonstrates superiority over the best SOTA segmentation model (MAnet [32]). Specifically, it outperforms MAnet by an IoU of 2.2%, a Recall of 2.88%, a Dice of 1.24%, and an F1-score of 1.24%. These results confirm the high efficiency and accuracy of the proposed model, making it superior not only to MAnet but also to the other six SOTA segmentation models. The very small standard deviation values indicate that our model performs well across all batches and proves the stability of the model’s performance. The results in Table 2 demonstrate the effectiveness of the proposed enhancements, reflecting clear improvements in the segmentation performance. The higher rates of Recall and precision indicate that our model can more effectively identify and classify objects in images and minimize the number of missed objects (false negatives) and misclassified objects (false positives). These findings indicate the value of our study and the development of the proposed approach; moreover, these results confirm the excellence of our model and indicate its potential for applications in various practical contexts where precise segmentation is critical. Therefore, the proposed model can provide a foundation for future research and development in image segmentation.

### 4.5. Ablation Study

In this study, we conducted a series of ablative experiments to identify the model’s most effective combination of hyperparameters and architectural components. To achieve this, we tested six different combinations, each representing a variation in critical components of the model. The results in Table 3 showed that our model achieved the best performance using the optimal combination of parameters. In particular, the highest values of IoU and F1-score metrics were obtained in this configuration. These metrics confirm the high accuracy and efficiency of the proposed model compared to alternative options.

The comparative analysis of Table 3 shows that the proposed model shows a significant improvement over other models in cardiac segmentation. In terms of the average IoU, the proposed model shows an improvement of 2.52% over M1, 2.58% over M2, 2.81% over M3, 4.9% over M4, 1.99% over M5, and 2.39% over M6. Regarding the average F1-score, the proposed model also significantly outperforms the other models: the improvement is 1.42% relative to M1, 1.45% relative to M2, 1.59% relative to M3, 2.8% relative to M4, 1.12% relative to M5, and 1.34% relative to M6.

These results tell us that the proposed model effectively utilizes the latest architectural design and training methods; this in turn facilitates the achievement of a higher accuracy in the object segmentation in images compared to existing standards.

## 5. Discussion

In this section, we present a comprehensive comparison of the proposed model with current (SOAT) techniques, analyze data from previous studies, and demonstrate the model’s ability to generalize across many diverse datasets. The results show that our model outperforms existing counterparts on several key measures, which in turn proves its effectiveness and generalizability in diverse settings and data types.

### 5.1. Generalization Results

#### 5.1.1. On Wound Images Database

**Figure 12:**
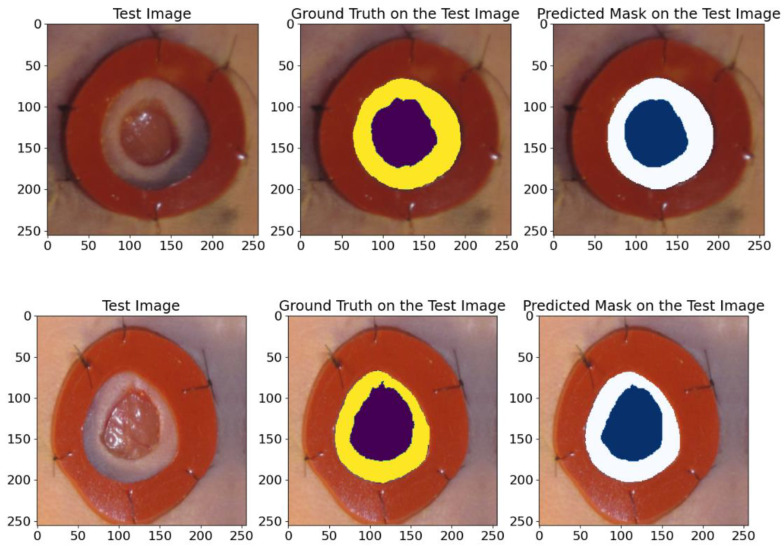
An example of images from the wound closure progress database. The visualization includes raw images, real ground truths, and model-predicted ground truths. The deep violet and the blue colors indicates the wounded areas, while the yellow and white indicate the areas around the wound.

The results of this experiment are presented in Table 4. They demonstrate that the proposed model achieved high average values for the IoU, Recall, Dice, precision, and F1-score metrics, which confirms its high efficiency in the segmentation task. Figure 12 shows various examples from the test dataset with the corresponding reference masks and the mask predictions obtained by the model. These examples illustrate the model’s ability to accurately predict masks corresponding to real anatomical structures.

The comparison in Figure 12 shows the high accuracy of the model in segmenting the wounds and the areas around them, confirming its applicability for clinical research and analysis.

#### 5.1.2. On the Kavsir-SEG Database

**Figure 13:**
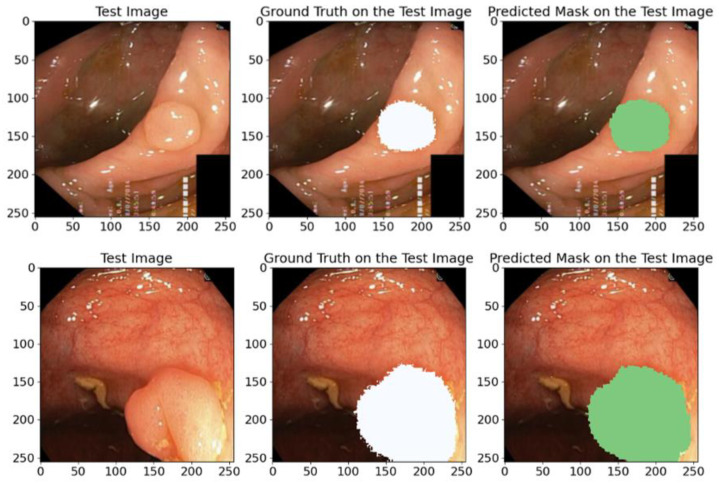
A visual example of random samples from the Kvasir-SEG test dataset with corresponding reference masks and predicted masks using our proposed model. White and green regions indicate the areas segmented as polyps. This comparison demonstrates the model’s high segmentation accuracy.

The validation results presented in Table 5 confirmed the high IoU and Dice coefficient achieved by the proposed model. These results indicate the model’s high efficiency and reliability in the polyp segmentation task. To further illustrate the model’s capabilities, we randomly chose samples from the test dataset and made predictions on their masks, as depicted in Figure 13. The visual comparisons of the predicted masks with the reference masks confirm the model’s ability to accurately segment polyps, which highlights its potential for clinical utility and improving diagnostic methods.

The predicted masks in Figure 13 illustrate the high IoU values and clearly show that the proposed model can accurately identify anatomical structures, which confirms its practical value and applicability in medical segmentation problems.

#### 5.1.3. On the CVC-Clinic Database

**Figure 14:**
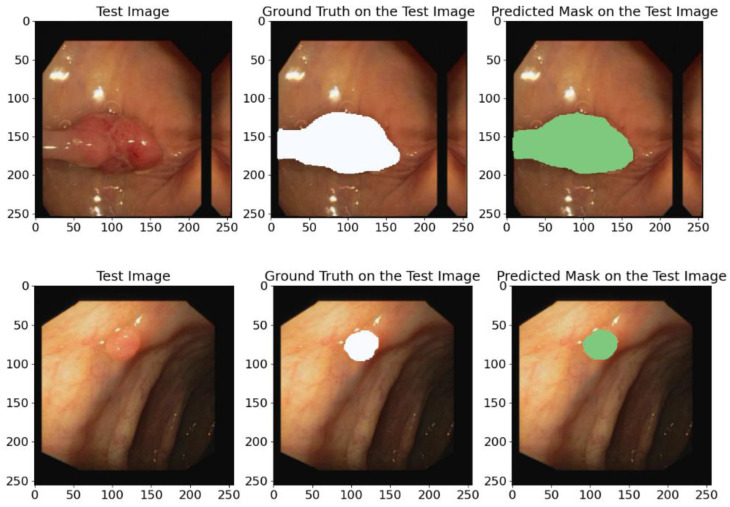
Diverse examples of random image instances from the CVC-ClinicDB test database containing the original annotations (GT) and the predicted annotations obtained using the proposed model. White and green regions correspond to the segmented polyps.

In this research study, we also utilize the CVC-ClinicDB database to test the generalization capability of our proposed model. The results of this experiment are presented in Table 6 and illustrated in Figure 14. We analyze these outcomes to evaluate and see how superior the effectiveness and generalization ability of our model is in identifying polyps and other colon pathologies.

Our model achieved the following average metric values: IoU—89.72%, Recall—93.80%, Dice coefficient—94.54%, Precision—95.40%, and F1-score—94.54%. These high scores further validate the model’s ability to generalize to new unseen data from other datasets.

The predicting ground truth masks in Figure 14 show that the proposed model demonstrates a high precision and confirms its ability to generalize to new data types and its effectiveness in identifying polyps and other colon pathologies.

#### 5.1.4. On ISIC 2017 Database

**Figure 15:**
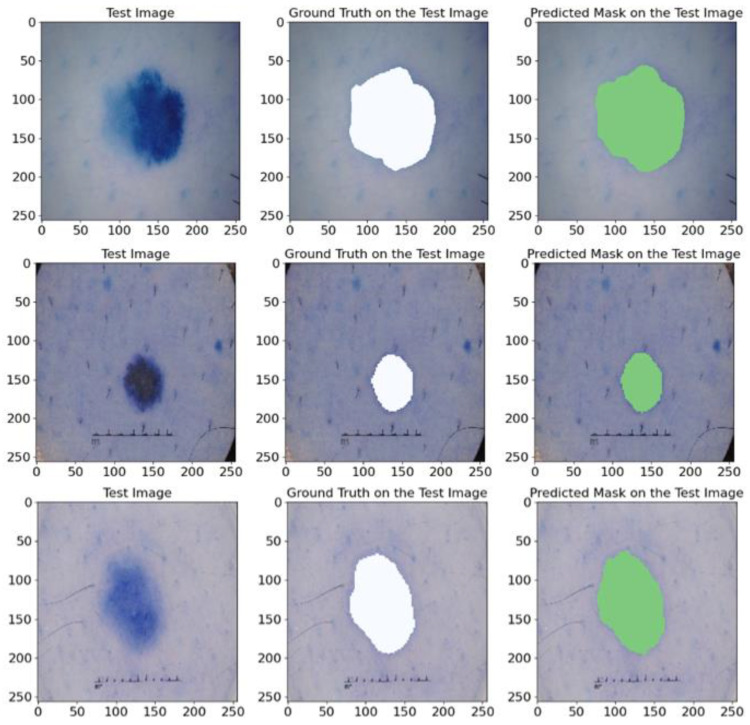
A visualization of the original image, the corresponding annotated mask, and the mask predicted using our model. The white and green regions represent the correctly segmented lesion areas.

To validate how our model effectively generalizes on the ISIC 2017 database, we utilized several stages. First, the images and their corresponding masks from ISIC 2017 were divided into training and test sets; then the model was fine-tuned and trained on the training set; after that, its performance was assessed on the test set. The outcomes showed that the model maintained a high level of precision on the training data and successfully generalized its predictions to new, previously unseen images. In Figure 15, we illustrate randomly selected test instances for a clear visualization. Figure 15 shows that the model demonstrated an exceptional precision in mask prediction and successfully identified the boundaries of skin lesions. This result verifies the efficiency and generalization of the model, which is especially relevant for clinical use and automated diagnostics.

Our experiments on the ISIC 2017 dataset demonstrate that the proposed model reaches various essential measurements that are described in Table 7. More specifically, the model achieved high average rates, including a mean IoU of 85.37%, a mean Recall of 91.63%, a mean Dice of 91.91%, a mean precision of 92.65%, and a mean F1-score of 91.91%, which further proves its ability to accurately and efficiently segment skin lesions in images.

Thus, the generalization experiment that we conducted on the ISIC 2017 dataset confirms the high efficiency of our proposed model in segmenting skin lesions, which makes it potentially useful for clinical utility and facilitates the automation of diagnostics.

### 5.2. Validation Results on ACDC 2017 Database

Table 8 shows the findings obtained when we validated our model on ACDC 2017 according to several factors, such as the average IoU and other metrics. The model showed high metrics results that verify its applicability and reliability. The average MIoU reached an 88.76% value, which indicates a high segmentation accuracy and tells us that the model effectively identifies and separates cardiac structures in MRI images. For other indicators, such as the Recall, Dice, Precision, and F1-score, the proposed model also shows high values, which further confirms its stability and reliability. The high results for all presented metrics confirm that the developed model is applicable for clinical studies and can be used to enhance cardiac MRI segmentation algorithms further. These findings highlight the model’s effectiveness in resolving segmentation issues and its potential for use in clinical settings.

**Figure 16:**
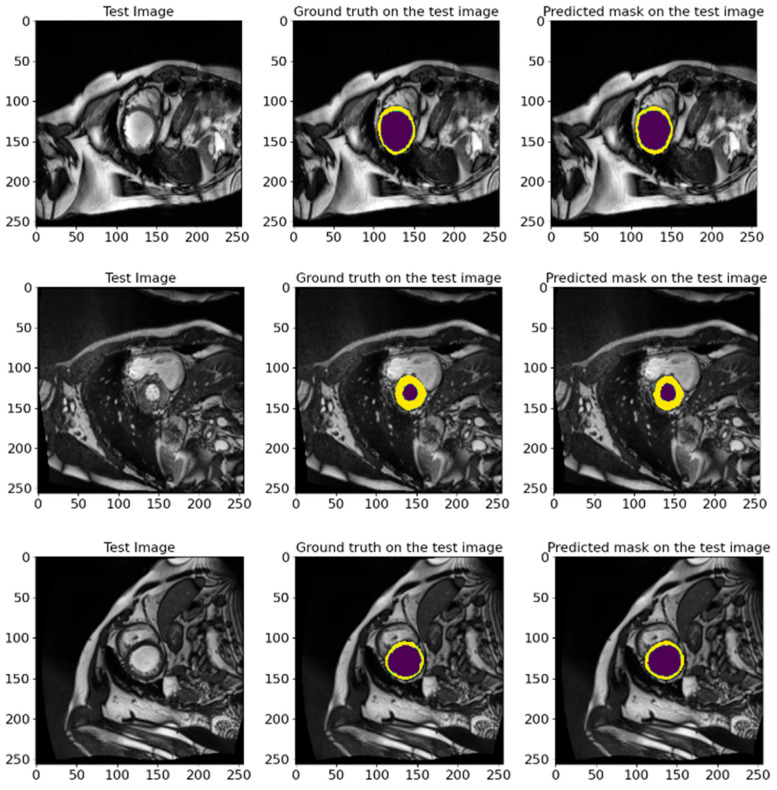
The visual presentation of random samples of images from the test set from the ACDC 2017 database, with the actual and predicted GT by the model. The visualization shows the comparison between the manual annotation conducted with experienced cardiologists and the segmentation results obtained using the developed model. The deep violet marks the LV regions, whereas yellow outlines the Myo. The high degree of agreement between predictions and actual labels authenticates the precision and reliability of our proposed model in segmenting cardiac MRI images.

In Figure 16, we show random samples from the test set, along with their GT masks and the predicted masks generated by the model. This visualization illustrates the comparison between the manual annotation conducted with the help of experienced cardiologists and the segmentation outcomes acquired using the developed model. The actual labels represent the true state of the segmented regions and are presented in each image for visual comparison. Looking at the predictions of the model, they indicate how accurately it can reproduce the contours and shapes of the left ventricle and myocardium. The comparison of the images in Figure 16 attests to the high accuracy of the model since the predicted segmentations almost entirely match the actual labels. It further highlights the model’s applicability in clinical settings and its ability to conduct the segmentation task on cardiac MRI images efficiently.

### 5.3. A Validation on an Unlabeled EMIDEC 2020 Database

**Figure 17:**
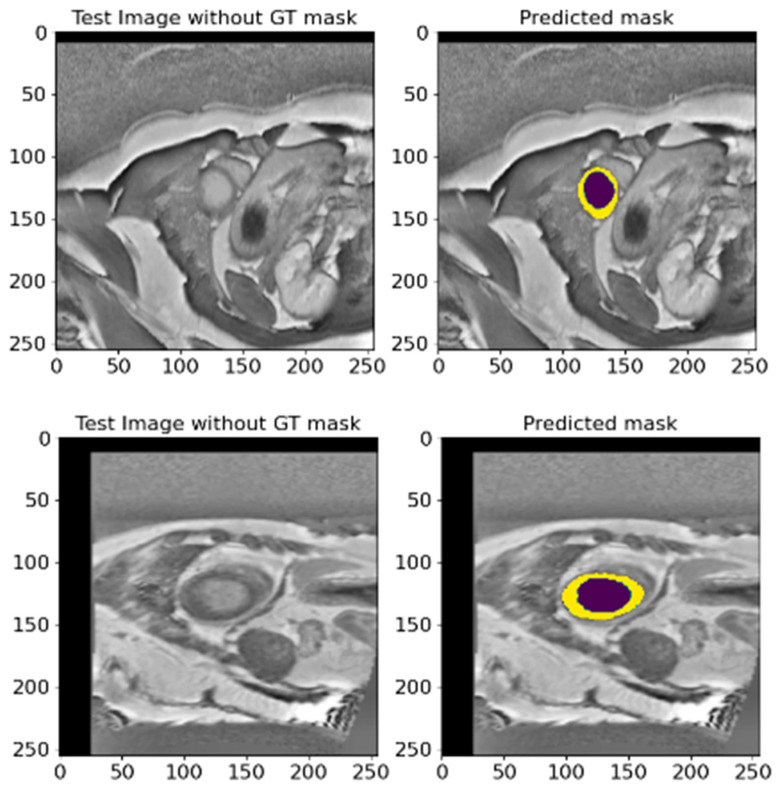
Two diverse instances of predicted masks for randomly selected images from the EMIDEC test set database without reference labels. This figure shows the original images and predicted masks obtained utilizing the proposed model. Deep violet signifies the LV, and yellow denotes the Myo region.

To evaluate the performance of the proposed model in the absence of reference masks, we carried out an ablation on 358 images from the EMIDEC 2020 [36] database. This test set does not contain a reference ground truth, so we used it to validate the model and check the generalization and applicability of the proposed method. We conducted this test because it is critical in determining how well our model can perform on new unseen data that does not have ground truth masks. This approach allowed us to test whether the model could maintain its optimal efficiency without relying on pre-labeled data. Figure 17 shows the results of mask predictions for randomly chosen images from this test set. These results demonstrate that the proposed model can generate precise masks corresponding to anatomical structures without reference labels. The model showed impressive results, indicating its high level of generalization and adaptability. This ability of the model to effectively deal with new and unseen data proves its potential practical value in actual clinical settings, where the availability of labeled data is not always guaranteed. These results strengthen our confidence that the proposed model can be applied to a wide range of medical segmentation tasks and provide an optimal efficiency and robustness even when working with unlabeled data.

Overall, this conducted experiment emphasizes the relevance and efficacy of the proposed segmentation model in demonstrating its generalizability and applicability across various settings. It makes it a valuable utility for medical research and practical applications and is capable of enhancing the quality and precision of medical image analyses.

### 5.4. Cross-Dataset Generalization: A Comparative Analysis

To rigorously assess the generalization capability of the proposed ResST-SEUNet++, we conducted a comparative evaluation across one cardiac and four non-cardiac medical image segmentation datasets. Building on our initial benchmarking comparative experiment on the EMIDEC dataset (Table 2), we identified the top three performing models—MAnet, ResUnet, and LinkNet—based on their performance across the utilized standard metrics. These models were subsequently selected as strong baselines for external comparison. As presented in Table 9, ResST-SEUNet++ consistently achieved a superior segmentation performance relative to these three high-performing models across all five external datasets. This consistent outperformance underscores the model’s robust generalization capacity and its adaptability to domain shifts beyond cardiac imaging. By restricting the comparison to top-tier models, we ensured both computational efficiency and a targeted evaluation framework, allowing for clearer insights into the effectiveness of the proposed architecture. This comparative analysis affirms the generalization strength of the proposed approach and further substantiates its potential for a broader clinical deployment across varied segmentation tasks.

### 5.5. Comparison with Previous Studies

The present study proposes a DL architecture for cardiac segmentation in short-axis MR images that demonstrated an exceptional performance compared to previous studies, which are elaborated in Table 10. Previously, Cui et al. [48] presented the Multi-Scale Attention-Guided U-Net technique, which achieved an IoU value of 75%. A study by Tan et al. [49] proposed a regression convolutional neural network that achieved an IoU of 77%. Similarly, Tran [50] developed a fully convolutional neural network for heart segmentation that achieved a mean IoU of 74% and a mean Recall of 83%. Khened et al. [51] applied multi-layer DenseNets and achieved an IoU of 74% and a Dice of 84%. The study by Wang et al. [52] used a dynamic pixel weighting technique that demonstrated IoU values of 70% and a Dice of 80%.

In more recent studies, such as Chen et al. [53], a high performance was achieved, characterized by mean IoU and mean Dice values of 83.89% and 91.04%, respectively. Da Silva et al. [54] developed a cascade approach for automatically segmenting cardiac structures, achieving a mean IoU, Dice, Recall, and F1-score of 80.89%, 86.88%, 87.48%, and 87.43%, respectively. In a new study, Al-antari et al. [55] achieved a mean IoU of 84.23%, a mean Recall of 85.24%, and a mean F1-score of 85.35% using the ResUNet model for cardiac segmentation. Compared to these related studies, the model proposed in this study shows significantly improved results, achieving an average IoU of 93.73%, an average Recall of 96.54%, an average Dice of 96.70%, an average precision of 96.86%, and an average F1-score of 96.70%. These results confirm the proposed architecture’s effectiveness and superiority over previous techniques, which opens up new opportunities for improving automatic cardiac segmentation in short-axis MRI images.

### 5.6. Explaining Model Decisions

**Figure 18:**
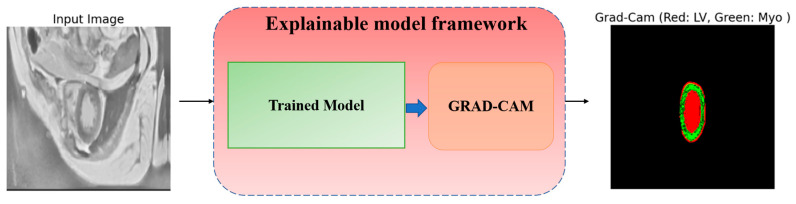
Explainable GRAD-CAM workflow from raw MRI input image to heatmap visualization.

To trust the findings and predictions attained by the model, we utilized a Grad-CAM technique, introduced in [56], to explain the reasoning behind the results and visualize the area of the input data that most contributed to the model’s decisions. Grad-CAM is a method that helps us understand how a neural network makes decisions. It shows us the regions of an image that the model considers crucial for its prediction and shows how and why a model makes specific predictions. Grad-CAM uses information from the model’s internal layers to highlight these regions. This is especially useful in medical problems where it is important to not only obtain a result, but also to understand how it was obtained. The visualized framework of the explainable model is illustrated in Figure 18.

**Figure 19:**
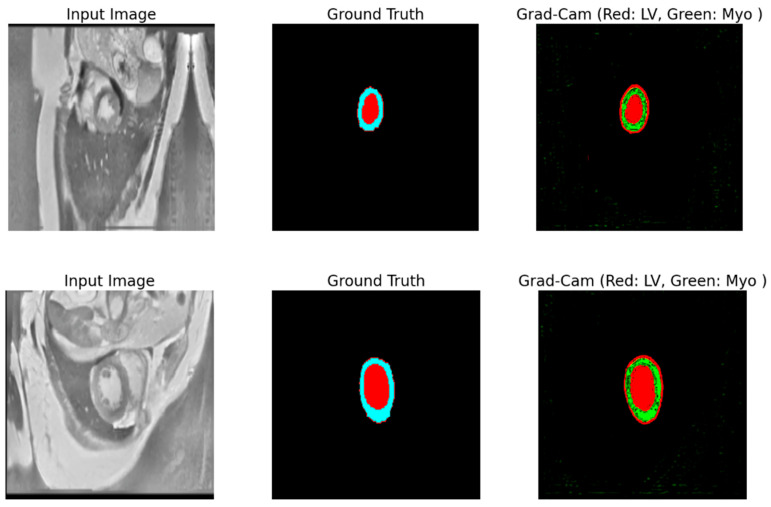
The visualization of Grad-CAM results. The left panel shows the input MRI images. The center panel highlights the ground truths, with red indicating the LV and light blue representing the Myo. The right panel illustrates Grad-CAM activations, visualizing the areas for the LV (Red) and Myo (Green) that the model focuses on during segmentation.

The process inside the framework is simply and straightforwardly detailed as follows: the input MRI raw image goes into the trained model, which predicts the outcome, then Grad-CAM pinpoints which parts (areas) of the image most influenced the predictions, and the heatmap highlights the crucial ones in the MRI image. In Figure 19, we visualize two distinct samples in three columns. The original image (left), which is an MRI slice of the heart region, serves as input to the model. The true markup (ground truth) (center) is a reference created by experts that shows where the LV (Red) and Myo (light blue) are. Grad-CAM overlays (right) show which areas the model paid attention to when analyzing the image. In this column, red areas are associated with the LV, while green areas are associated with the Myo. Grad-CAM shows that the model correctly identifies areas corresponding to the LV and Myo. They almost completely coincide with the true markings. From analyzing Figure 19, we see two crucial facts:

- The increased intensity of overlays, to see crucial areas, confirms that the model confidently works with key structures.
- The model clearly distinguishes the boundaries between the LV and Myo, even if they are located close to each other.

These results show that the model works accurately and reliably. It not only correctly recognizes crucial structures, but also makes it understandable for a person. This is especially important in medicine, where the health and lives of patients depend on such decisions. Thus, utilizing Grad-CAM in this research not only helps validate the model but also shows where it can be improved. For example, refining the activation boundaries can make the segmentation even more accurate, especially in complex cases. Consequently, the model demonstrates an excellent performance in segmenting cardiac structures, and the use of Grad-CAM enhances the interpretability, increasing the confidence in the results. This makes the model valuable for both clinical applications and further research.

### 5.7. Limitations

Despite the good results achieved, this study has some limitations that must be considered when interpreting the data obtained and planning further research. Firstly, the size and diversity of the dataset may limit the model’s overall applicability. Our study utilized data collected from a limited number of sources and under certain conditions. It may not entirely reflect all the variability in anatomical and pathological characteristics that may be experienced in clinical practice. Thus, including more diverse and representative datasets in the future may further enhance the generalization capability of the model.

Secondly, the selected architecture of the model and the hyperparameters have not been optimized for all types of data and segmentation tasks. However, even with meticulous adjustments, alternative designs or combinations of hyperparameters may exhibit a superior performance. Thus, additional testing with different structures and optimization strategies can enhance the selection of the most efficient ways. Also, the model was trained and tested in a controlled setting, which might not be exactly like a real clinical setting, where the image quality and other outside factors can change a lot; therefore, moving from the lab to real-life clinical practice needs more study and the validation of the data collected in various medical settings and institutions.

Lastly, creating and utilizing DL models in clinical practice needs to take ethics and legal issues into consideration, for instance, to keep patients’ data safe and ensuring these algorithms are explicit. Thus, it is relevant to conduct more investigations and discourse on these issues.

Therefore, even though this study has good results and potential, some issues need to be considered. These problems need to be fixed in future studies so that DL models can better accurately and reliably split heart parts for usage in actual life.

### 5.8. Future Work

The present work employed deep learning models to segment the LV and Myo. Notwithstanding the obtained results, there exist certain domains in which more investigation can greatly enhance the quality and precision of the segmentation.

Moving forward, there are intentions to broaden the scope of segmentation to include additional structural components of the heart, particularly the right ventricle [57], myocardial infarction [58], and atria. This will facilitate the development of more extensive models capable of precisely analyzing the integrated anatomy and function of the heart. Having an enhanced precision in modeling different diseased states of the heart, such as the formation of scar tissue after a myocardial infarction, will provide a more comprehensive diagnosis and treatment planning.

Furthermore, the utilization of advanced deep learning models, such as neural network ensembles, Vision Transformer (ViT), and multi-modal techniques, has the potential to enhance the overall precision and resilience of the model. The integration of transfer learning and continuous learning approaches enables models to dynamically adjust to new data and enhance their predictions as new information is acquired. The integration of data from many sources, including magnetic resonance imaging (MRI) and computed tomography (CT), might enhance the segmentation quality by providing further information.

Therefore, future efforts should focus on enhancing the capabilities of the models, enhancing their precision and dependability, and incorporating them into feasible medical operations. These research areas will contribute to the development of more precise and dependable instruments for the diagnosis and treatment of cardiovascular illnesses, thus resulting in an enhanced quality of care and patient well-being.

## 6. Conclusions

This study presented a comprehensive LV and Myo segmentation method based on DL models. Our method involves image pre-processing utilizing CLAHE and bilateral filtering to enhance the quality of the input data. This pre-processing helps enhance the contrast and eliminate noise, hence improving the subsequent segmentation quality.

The model we offer demonstrates a far superior performance compared to established designs, including PAN, ResUnet, ResUnet++, MAnet, Linknet, and PSPnet. Our analysis demonstrated that the suggested method yields a superior accuracy and reliability in segmentation, as indicated by the achieved average values of the IoU (93.73%), Recall (96.54%), Dice (96.70%), precision (96.86%), and F1-score (96.70%). The superior performance on these criteria demonstrates the model’s exceptional capacity to differentiate and precisely segment cardiac anatomical components. We have shown that our suggested model exhibits excellent average IoU values on five other databases, therefore confirming its adaptability and efficacy in many application domains. The average IoU values were 88.78% on the ACDC 2017 database, 92.95% on the Wound Closure Database, 86.04% on the Kvasir-SEG database, 89.72% on the CVC-ClinicDB database, and 85.37% on the ISIC 2017 database. These results demonstrate the model’s ability to adapt and maintain a high segmentation precision in a variety of medical and other applications, which proves its potential applicability to a wide range of image analysis tasks. Our work highlights the significance of utilizing advanced image pre-processing techniques and optimized DL architectures to achieve a superior performance in medical segmentation tasks.

We can say that our suggested method works better than other methods and creates new ways to use DL in medical detection and heart image analysis. The results keep the hope alive for more study in this area, such as making models that are more complex and adaptive so they can handle a wider range of clinical situations. By making it easier to diagnose and treat cardiovascular illnesses, putting these types of models to use in clinical practice can greatly enhance the level of treatment.

## Figures and Tables

**Table 1:** Performance rates of the proposed segmentation model.

Model	Mean IoU (%) ± SD	Mean Recall (%) ± SD	Mean Dice (%) ± SD	Mean Precision (%) ± SD	Mean F1-Score (%) ± SD	Epochs	Params (M)	LR/Batch
Proposed	93.73 ± 0.0137	96.54 ± 0.0081	96.70 ± 0.0076	96.86 ± 0.0074	96.70 ± 0.0076	100	53	0.0001/8

**Table 2:** Performance comparison of different SOTA deep segmentation models.

Model	Mean IoU (%) ± SD	Mean Recall (%) ± SD	Mean Dice (%) ± SD	Mean Precision (%) ± SD	Mean F1-Score (%) ± SD	LR/Batch
ResUnet++ [34]	91.20 ± 0.0074	93.79 ± 0.0052	95.28 ± 0.0043	97.00 ± 0.0041	95.28 ± 0.043	0.0001/8
PAN [31]	90.90 ± 0.0095	93.49 ± 0.0066	95.10 ± 0.0055	96.98 ± 0.0054	95.10 ± 0.0055	0.0001/8
ResUnet [33]	91.33 ± 0.0091	93.49 ± 0.0073	95.35 ± 0.0053	97.47 ± 0.0045	95.35 ± 0.0053	0.0001/8
LinkNet [29]	91.29 ± 0.0099	93.75 ± 0.0069	95.33 ± 0.0057	97.12 ± 0.0054	95.33 ± 0.0057	0.0001/8
MAnet [32]	91.53 ± 0.0078	93.66 ± 0.0059	95.46 ± 0.0044	**97.52 ± 0.0041**	95.46 ± 0.0044	0.0001/8
PSPNet [30]	90.69 ± 0.0103	93.38 ± 0.0074	94.98 ± 0.0060	96.85 ± 0.0058	94.98 ± 0.0060	0.0001/8
Proposed	**93.73 ± 0.0137**	**96.54 ± 0.0081**	**96.70 ± 0.0076**	96.86 ± 0.0074	**96.70 ± 0.0076**	0.0001/8

The highest values in each column are highlighted in bold.

**Table 3:** Comparative results of ablation experiments.

Model	Mean IoU (%) ± SD	Mean F1-Score (%) ± SD
Unet++ with Resnet34 (M1)	91.21 ± 0.0082	95.28 ± 0.0047
Unet++ with Resnet34 and SE decoder attention (M2)	91.15 ± 0.0078	95.25 ± 0.0045
Unet++ with Resnet 34 and scSE decoder attention (M3)	90.92 ± 0.0092	95.11 ± 0.0053
Unet++ alone (M4)	88.83 ± 0.0130	93.90 ± 0.0078
Unet++ with resnest50d and scSE decoder attention (M5)	91.74 ± 0.0077	95.58 ± 0.0044
Proposed without CLAHE (M6)	91.34 ± 0.0070	95.36 ± 0.0040
Proposed (Unet++ with resnest50d and SE decoder attention)	**93.73** ± **0.0137**	**96.70** ± **0.0076**

**M1–6** is an abbreviation for the name of the model combination.

**Table 4:** Performance metrics of the proposed model on the Wound Closure Database.

Model	Mean IoU (%) ± SD	Mean Recall (%) ± SD	Mean Dice (%) ± SD	Mean Precision (%) ± SD	Mean F1-Score (%) ± SD	Epochs	Params (M)	LR/Batch
Proposed	92.95 ± 0.0101	95.45 ± 0.0085	96.27 ± 0.0057	97.14 ± 0.0042	96.27 ± 0.0057	100	53	0.0001/8

**Table 5:** Primary performance metrics of our proposed model on the Kavsir-SEG database.

Model	Mean IoU (%) ± SD	Mean Recall (%) ± SD	Mean Dice (%) ± SD	Precision (%) ± SD	Mean F1-Score (%) ± SD	Epochs	Params (M)	LR/Batch
Proposed	86.04 ± 0.0737	91.26 ± 0.0625	92.32 ± 0.0451	93.74 ± 0.0493	92.32 ± 0.0451	100	53	0.0001/8

**Table 6:** Results of testing the generalization ability of the proposed model on the CVC-Clinic database.

Model	Mean IoU (%) ± SD	Mean Recall (%) ± SD	Mean Dice (%) ± SD	Mean Precision (%) ± SD	Mean F1-Score (%) ± SD	Epochs	Params (M)	LR/Batch
Proposed	89.72 ± 0.0345	93.80 ± 0.0339	94.54 ± 0.0195	95.40 ± 0.0206	94.54 ± 0.0195	100	53	0.0001/8

**Table 7:** Detailed performance metrics (%) of the proposed model on the ISIC 2017 database.

Model	Mean IoU (%) ± SD	Mean Recall (%) ± SD	Mean Dice (%) ± SD	Mean Precision (%) ± SD	Mean F1-Score (%) ± SD	Epochs	Params (M)	LR/Batch
Proposed	85.37 ± 0.0689	91.63 ± 0.0529	91.91 ± 0.0430	92.65 ± 0.0629	91.91 ± 0.0430	100	53	0.0001/8

**Table 8:** The performance metrics of validating the proposed model on the ACDC database.

Model	Mean IoU (%) ± SD	Mean Recall (%) ± SD	Mean Dice (%) ± SD	Mean Precision (%) ± SD	Mean F1-Score (%) ± SD	Epochs	Params (M)	LR
Proposed	88.78 ± 0.0175	93.39 ± 0.0138	93.85 ± 0.0106	94.38 ± 0.0101	93.85 ± 0.0106	100	53	0.0001

**Table 9:** Generalization performance comparison of ResST-SEUNet++ and top baseline models across five segmentation datasets.

Database	Model	Mean IoU (%)	Mean Recall (%)	Mean Dice (%)	Mean Precision (%)	Mean F1-Score (%)
Wound Closure	MAnet	92.20 ± 0.0180	94.77 ± 0.0178	95.83 ± 0.0232	97.01 ± 0.0010	95.83 ± 0.0232
	ResUnet	92.06 ± 0.0213	94.69 ± 0.0185	95.75 ± 0.0133	96.92 ± 0.0067	95.75 ± 0.0133
	LinkNET	92.26 ± 0.0134	94.87 ± 0.0118	95.88 ± 0.0077	96.98 ± 0.0046	95.88 ± 0.0077
	Proposed	**92.95 ± 0.0101**	**95.45 ± 0.0085**	**96.27 ± 0.0057**	**97.14 ± 0.0042**	**96.27 ± 0.0057**
Kavsir-SEG	MAnet	80.32 ± 0.0812	89.99 ± 0.0832	88.85 ± 0.0516	88.60 ± 0.0705	88.85 ± 0.0516
	ResUnet	83.25 ± 0.0669	89.64 ± 0.0627	90.71 ± 0.0413	92.18 ± 0.0495	90.71 ± 0.0413
	LinkNET	83.30 ± 0.0823	89.63 ± 0.0757	90.65 ± 0.0520	92.27 ± 0.0594	90.65 ± 0.0520
	Proposed	**86.04 ± 0.0737**	**91.26 ± 0.0625**	**92.32 ± 0.0451**	**93.74 ± 0.0493**	**92.32 ± 0.0451**
CVC-Clinic	MAnet	88.81 ± 0.0454	92.78 ± 0.0437	94.01 ± 0.0262	95.41 ± 0.0215	94.01 ± 0.0262
	ResUnet	88.24 ± 0.0503	92.04 ± 0.0523	93.67 ± 0.0295	**95.54 ± 0.0155**	93.67 ± 0.0295
	LinkNET	88.79 ± 0.0527	93.42 ± 0.0367	93.97 ± 0.0327	94.63 ± 0.0401	93.97 ± 0.0327
	Proposed	**89.72 ± 0.0345**	**93.80 ± 0.0339**	**94.54 ± 0.0195**	95.40 ± 0.0206	**94.54 ± 0.0195**
ISIC 2017	MAnet	83.39 ± 0.0702	90.04 ± 0.0695	90.78 ± 0.0433	91.92 ± 0.0432	90.78 ± 0.0433
	ResUnet	83.13 ± 0.0747	87.73 ± 0.0743	90.59 ± 0.0478	94.08 ± 0.0410	90.59 ± 0.0478
	LinkNET	83.30 ± 0.0801	87.97 ± 0.0907	90.67 ± 0.0518	**94.21 ± 0.0296**	90.67 ± 0.0518
	Proposed	**85.37 ± 0.0689**	**91.63 ± 0.0529**	**91.91 ± 0.0430**	92.65 ± 0.0629	**91.91 ± 0.0430**
ACDC 2017	MAnet	87.87 ± 0.0150	92.15 ± 0.0147	93.22 ± 0.0171	**94.56 ± 0.046**	93.22 ± 0.0171
	ResUnet	87.56 ± 0.0682	91.95 ± 0.0713	92.73 ± 0.0704	93.65 ± 0.0711	92.73 ± 0.0704
	LinkNET	87.22 ± 0.0676	91.28 ± 0.0706	92.52 ± 0.0701	93.98 ± 0.0717	92.52 ± 0.0701
	Proposed	**88.78 ± 0.0175**	**93.39 ± 0.0138**	**93.85 ± 0.0106**	94.38 ± 0.0101	**93.85 ± 0.0106**

The highest values are shown in bold.

**Table 10:** Comparison of performance rates of different cardiac segmentation methods on short-axis MR images.

Method	Dataset	Methods	Mean IoU (%)	Mean Dice (%)	Mean Recall (%)	Mean F1-Score (%)
Cui et al. [48]	LVSC	An Attention U-Net with image pyramids and Focal Tversky Loss for cardiac MRI segmentation.	75	–	–	–
Tan et al. [49]	LVSC	Dual-network model for LV segmentation via center localization and polar border regression.	77	–	–	–
Tran et al. [50]	LVSC	A fully convolutional network (FCN) for end-to-end, pixel-level segmentation of cardiac MRI images.	74	–	83	–
Khened et al. [51]	LV-2011	DenseNet-based FCN using Inception modules and dual loss for efficient cardiac segmentation.	74	84	–	–
Wang et al. [52]	LV DETERMINE dataset	Dynamic pixel-wise weighted FCN with scale-invariant features for automatic LV MRI segmentation.	70	80	–	–
Chen et al. [53]	ACDC-2017	PH-Mamba boosts segmentation by preheating state space models with contextual cues and compensation.	83.89	91.04	–	–
Silva et al. [54]	ACDC-2017	A U-Net identifies regions of interest, a custom FCN segments cardiac structures, followed by refinement via a reconstruction module.	80.89	86.88	87.48	87.43
Al-antari et al. [55]	EMIDEC 2020	Multi-class cardiac segmentation using enhanced MRI and an adapted ResUNet.	84.23	–	85.24	85.35
Proposed	EMIDEC 2020	CLAHE-enhanced MRI and ResST-SEUNet++, a deep learning model integrating a ResNetSt encoder with SE-attention mechanisms in the UNet++ decoder, for improved cardiac image segmentation.	**93.73**	**96.70**	**96.54**	**96.70**

Values in bold represent the highest scores.

## Data Availability

All six datasets used in this study are publicly available.
